# Molecular characterization of feline caliciviruses isolated from several adult cats with atypical infection showing severe flu-like symptoms on a remote island in Ehime, Japan

**DOI:** 10.1016/j.virusres.2025.199535

**Published:** 2025-01-30

**Authors:** Yuki Nishisaka, Hikaru Fujii, Fumiko Ono, Sho Kadekaru, Hiroyuki Kogiku, Yumi Une, Shione Takeguchi, Naomi Ohta, Masumi Eto, Chiharu Takeuchi, Seigou Takeuchi, Tetsuko Miki, Akihiko Tokuda, Keiko Ookawa, Yukinobu Tohya, Keita Ishijima, Akiko Okutani, Ken Maeda, Shumpei Watanabe, Shigeru Morikawa

**Affiliations:** a Department of Microbiology, Faculty of Veterinary Medicine, Okayama University of Science, Imabari, Ehime, Japan; b Laboratory Animal Science, Faculty of Veterinary Medicine, Okayama University of Science, Imabari, Ehime, Japan; c Laboratory of Veterinary Pathology, Faculty of Veterinary Medicine, Okayama University of Science, Imabari, Ehime, Japan; d Department of Epidemiology, Faculty of Veterinary Medicine, Okayama University of Science, Imabari, Ehime, Japan; e Biochemistry Unit, Faculty of Veterinary Medicine, Okayama University of Science, Imabari, Ehime, Japan; f OzuMorino Animal Hospital, Ozu, Ehime, Japan; g Daktari Animal Hospital Kansai Medical Center, Sakai, Osaka, Japan; h Ryunosuke Animal Hospital, Kumamoto, Kumamoto, Japan; i Laboratory of Veterinary Microbiology, Department of Veterinary Medicine, College of Bioresource Sciences, Nihon University, Fujisawa, Kanagawa, Japan; j Department of Veterinary Science, National Institute of Infectious Diseases, Shinjuku, Tokyo, Japan

**Keywords:** Feline calicivirus, Virulent systemic feline calicivirus, VP1, Feline infectious disease, Upper respiratory tract disease, Severe flu-like symptom, Anemia

## Abstract

In November 2020, a volunteer group reported an outbreak of an infectious disease with a high fatality rate and flu-like symptoms among stray cats in Aoshima, a remote island in Ehime, Japan. Nine adult cats with severe symptoms were hospitalized. Feline calicivirus (FCV) was isolated from pharyngeal swabs of six hospitalized cats. An outbreak of virulent systemic FCV (VS-FCV) infection was initially suspected because of obvious flu-like symptoms in adult cats; however, no symptoms typically associated with VS-FCV, such as skin ulcers on the limbs, edema, or viremia, were observed. Notably, two of the hospitalized cats that showed severe disease had diarrhea and anemia, and died or had a prolonged illness. These cases reveal atypical symptoms of FCV infection that have not been previously reported. We further isolated typical strains from western Japan (Osaka, Kumamoto, and Ehime) and analyzed the viral genes along with virulent strains from Aoshima. Phylogenetic analysis showed that the Aoshima strain formed a new lineage distinct from known FCVs. The Aoshima strains isolated in the initial outbreak before December 5, 2020, and those isolated after the end of the outbreak, which are suspected pathogenic and typical non-pathogenic strains, respectively, were located in the same cluster and shown to be very similar in sequence. The virulent Aoshima strain, which causes atypical FCV infections in cats, may have been derived by acquiring several mutations from a typical strain that chronically infects cats on a remote island.

## Highlights

• Epidemic of atypical Feline calicivirus (FCV) in Aoshima Island, Japan.
• Adult cats showed severe flu-like symptoms rarely accompanied by anemia and diarrhea.
• Aoshima virulent strain likely mutated from typical FCV.
• Aoshima virulent strain showed no serological cross-reactivity with an Osaka isolate.
• Implications for FCV pathogenicity in cats.

## 1 Introduction

Feline calicivirus (FCV) belongs to the genus *Vesivirus* the family *Caliciviridae* and contains a positive-sense RNA genome (approximately 7.7 kb), packaged within a non-enveloped capsid (Green, 2013). Its genome consists of three open reading frames (ORFs). ORF1 encodes a 200-kDa polyprotein that is processed by a viral protease (Pro) into six non-structural proteins, NS1 to NS6/7 (Alhatlani et al., 2015; Hofmann-Lehmann et al., 2022; Radford et al., 2007). ORF2 encodes a 73-kDa capsid precursor protein that is post-translationally processed to yield the 60-kDa mature capsid protein, VP1, as well as a 14-kDa leader of the capsid (LC) that is associated with establishment of cytopathic effect (CPE) and apoptosis induction (Abente et al., 2013; Barrera-Vázquez et al., 2019; Peñaflor-Téllez et al., 2022). ORF3 encodes the minor structural protein VP2. VP2 forms a portal-like assembly during early infection, releasing the viral genome through the endosomal membrane into the host cell cytoplasm (Conley et al., 2019). In addition to the genome RNA, a subgenomic RNA coterminal with 3′-terminal of the genomic RNA is also transcribed in the FCV-infected cell. The 5ʹ ends of genomic and subgenomic RNAs are linked to VPg without a cap structure and the 3ʹ ends are polyadenylated. (Wei et al., 2024). Most FCV strains belong to a single serotype because they are sufficiently antigenically related to induce some degree of cross-protection (Coyne et al., 2012; Geissler et al., 1997; Glenn et al., 1999). However, owing to antigenic differences among FCV isolates, a vaccine capable of preventing infection with all FCV isolates has not yet been developed (Poulet et al., 2000).

In addition to feline herpesvirus type 1 (FHV-1), FCV is a common cause of acute upper respiratory tract disease in cats worldwide (Radford et al., 2007). Cats infected with FCV commonly show no clinical symptoms or develop mild upper respiratory disease with oral ulcers (Radford et al., 2007). FCV infection can cause severe pneumonia in kittens, but rarely in adult cats (Slaviero et al., 2021). Similarly, although rare, limping may be associated with the infections (Dawson et al., 1994). In addition, FCV persists in cats even after recovery from symptoms, and cats become carriers of the virus (Green, 2013).

In 1998, an outbreak of virulent systemic feline calicivirus (VS-FCV), which causes systemic diseases, such as jaundice and edema in cats, was first reported in California, USA (Pedersen et al., 2000). During the outbreak, systemic symptoms were observed in adult cats and deaths were reported. Recently, sporadic outbreaks of VS-FCV and suspected VS-FCV infections were reported in Europe, Asia, and the United States (Bordicchia et al., 2021; Caringella et al., 2019; Guo et al., 2018; Ossiboff et al., 2007; Pedersen et al., 2000; Schorr-Evans et al., 2003). Although one field isolate was reported to show highly pathogenic symptoms in experimental infections of cats in Japan (Ohe et al., 2008), no outbreaks of epidemics suspected of VS-FCV infection have been reported to date.

In November 2020, several adult cats were found on Aoshima, a remote island in the Ehime Prefecture, Japan, where approximately 100 cats had become strays and showed severe flu-like symptoms. This suggests that the infection was spreading among cats living on the island. Cats with severe symptoms were transported by volunteers (an island cat protection group) to a veterinary hospital on the main island of Shikoku for treatment.

To identify the etiologic agent, pharyngeal swabs were taken from cats showing severe common flu-like symptoms, including symptoms suggestive of VS-FCV disease. Subsequently, we isolated viruses from the pharyngeal samples and attempted genetic and serological analyses of the isolated viruses.

## 2 Materials and methods

### 2.1 Cell lines and viruses

Feline kidney-derived and feline fetal-derived cells, CRFK and Fcwf-4 cells, were maintained in Dulbecco's Modified Eagle Medium (DMEM) (Nacalai Tesque, Kyoto, Japan) supplemented with 5% fetal bovine serum (FBS, DMEM-5FBS; Gibco, Waltham, MA, USA), and Penicillin-Streptomycin Mixed Solution (P/S solution; 50 units and 50 µg/ml, respectively; Nacalai Tesque). Vero cells stably expressing canine signaling lymphocytic activation molecules [Vero/DogSLAM cells (Seki et al., 2003)] were maintained in DMEM supplemented with 10% FBS (DMEM-10FBS) and P/S solution. The FCV vaccine strain, F9, was purchased from ATCC (VR-782) and propagated in CRFK cells. Infection titers in the CRFK cells of a group of FCV strains, including the F9 strain and other field strains isolated in this study, were determined using a standard 50% tissue culture infectious dose (TCID_50_) assay (Makino et al., 2006).

### 2.2 Virus isolation and growth

The virus inoculum was prepared with the DMEM-5FBS containing antibiotic cocktail solution [P/S solution, 50 µg/ml gentamicin (Nacalai Tesque), 2.5 µg/ml amphotericin B (Nacalai Tesque), and 50 µg/ml kanamycin (Nacalai Tesque)] as vehicle solution. One-half milliliter of the vehicle solution was added to a 2-ml tube, and the pharyngeal cotton swab was immersed into the tube and suspended. The suspension was centrifuged at 9390 × *g*, 10 min, 4 °C using a setting without operating brake, and the resultant supernatant was collected in a new 2-ml tube. The same centrifugation process was repeated thrice, and the resulting supernatant was used as the inoculum for virus isolation. The remainder of the inoculum was stored at −80 °C. Cultured cells (CRFK, Fcwf-4, or Vero/DogSLAM) seeded in six-well plates were infected with 200-µl of the above virus inoculum and allowed to adsorb for 1 h. Mock infection was performed with 200 µl/well of growth medium. After adsorption, the virus inoculum was removed, and 2 ml of fresh growth medium was added and incubated at 37 °C with 5% CO_2_.

When the virus was isolated by observing CPE, 10 µl of infectious culture supernatant was mixed with 3 ml of the DMEM-5FBS containing antibiotic cocktail, transferred to CRFK cells prepared in T75 flasks, and allowed to adsorb for 1 h at 37 °C. The inoculum was then removed and 13 ml of DMEM-5FBS was added and incubated at 37 °C. After 10–24 h, CPE was confirmed, following centrifugation at 850 × *g* for 10 min at 4 °C, the culture supernatant was collected, and the resulting supernatant was harvested as working stock and stored at −80 °C.

### 2.3 RNA extraction and viral gene detection

Total RNA was extracted from 140 µl of virus stock solutions using the QIAamp Viral RNA Mini Kit (Qiagen, Hilden, Germany) according to the manufacturer's protocol. cDNA was synthesized using the SuperScript III or IV First-Strand Synthesis System kit (Invitrogen, Waltham, MA, USA) with random primers according to the manufacturer's protocol. Then, PCR was performed using reported consensus primers targeting the VP1 gene of FCV (Cali1 and Cali4) (Supplementary Table S1) or feline parvovirus (FPV) (555F: 5ʹ-AGGAAGATATCCAGAAGGA-3ʹ and 555R: 5ʹ- GGTGCTAGTTGATATGTAATAAACA-3ʹ) (Buonavoglia et al., 2001; Marsilio et al., 2005). GoTaq DNA polymerase (Promega, Madison, WI, USA) was used for DNA extension during the PCR.

### 2.4 Detection of pathogen gene fragments in pooled samples by deep sequencing

After preparing pooled RNA by mixing the total RNA extracted from serum or pharyngeal swabs as described above, cDNA synthesis was performed using the NEBNext® Ultra™ II RNA Library Prep Kit for Illumina® (New England Biolabs, Ipswich, MA, USA). Then, DNA libraries were constructed using the ThruPLEX® DNA-Seq and DNA Unique Dual Index Kits (Takara Bio, Shiga, Japan). The resulting libraries were submitted to a sequencing service with Illumina NovaSeq (Illumina, San Diego, CA, USA) by Takara Bio Company. BWA-MEM 2 (Vasimuddin et al., 2019) and SAMtools (Li et al., 2009) were used to remove host-derived reads. De novo assembly of the obtained reads was performed using SPAdes Genome Assembler 3.15.2 (Bankevich et al., 2012), after the obtained sequences or contigs were classified using Kraken 2 (Wood et al., 2019) and BLAST (Camacho et al., 2009).

### 2.5 Determination of nucleotide sequence of FCV isolates

Primers for direct sequencing of the FCV isolates were initially designed based on the sequence of the F9 strain (Supplementary Table S1). RNA extraction and cDNA synthesis from the viral stock were performed as described in Section 2.3. PCR was performed using DNA polymerase, GoTaq Master Mix (Promega) or Q5 Hot Start High-Fidelity 2 × Master Mix (New England Biolabs). Synthesized cDNA (2 µl), 12.5 µl of GoTaq Master Mix or Q5 Master Mix, 1.25 µl each of forward and reverse primers, and 8 µl of distilled water were mixed, and PCR reactions were performed according to the manufacturer's protocol. The PCR products were electrophoresed on a 1% agarose gel and the amplified DNA fragments were excised and purified using the NucleoSpin Gel and PCR Clean-up Kit (Macherey-Nagel, Düren, Germany). Purified DNA was mixed with forward or reverse primers and sequenced (by Eurofins Genomics Tokyo, Japan). Based on the obtained fragment sequences, primers for direct sequencing were designed (Supplementary Table S1). PCR and direct sequencing were performed using the designed primers.

To determine the full-length genome or *VP1* gene of the FCV isolates, deep sequencing was performed using the MiSeq system (Illumina). Virus RNA of each strain was extracted from 250 µl of virus stock solutions using ISOGEN-LS (Nippon Gene, Tokyo, Japan) according to the manufacturer's protocol. Using the extracted RNA, synthesis of cDNA and double-stranded DNA, and sequence-independent single-primer amplification (SISPA) were conducted as previously described (Goya et al., 2018). After amplification of the first cDNA library with the SISPA primer, the DNA concentration of the resulting PCR products was determined using a Qubit (Thermo Fisher Scientific, Waltham, MA, USA). The second sample library was prepared for the MiSeq run using the Nextera XT DNA Library Prep Kit and the Nextera XT index kit-24 indexes (Illumina). The prepared DNA library was purified using Ampure XP (Beckman Coulter, Brea, CA, USA; 0.8X ratio), and its quality or DNA size distribution was evaluated using TapeStation 2200 (Agilent Technologies, Santa Clara, CA, USA). The sample pool to be loaded into the MiSeq v2 Reagent Kit (Illumina) was prepared by mixing the DNA libraries. The MiSeq Pooling Calculator program provided by Illumina was used to calculate the amount of each specimen added during pool preparation. For FCV isolates from Kotori and Scarlet (Table 1), the extracted RNAs was subjected to deep sequencing and analyzed by Genome Read Co. (Kagawa, Japan) using a DNBSEQ-G400RS system (MGI Tech, Shenzhen, China). To determine the 5ʹ-end sequence of FCV, we designed the forward primer F9–1f based on the highly conserved sequence in the region of the first few dozen bases of FCV (Supplementary Table S1) (Guo et al., 2018). We also designed several reverse primers based on the nucleotide sequence of strain F9 (Supplementary Table S1) and performed PCR using the primer set with F9–1f to determine the nucleotide sequence of the 5ʹ-end side of FCV. To determine the 3ʹ-end sequence of the FCV, a nested PCR was performed using a pair of primer specific for the FCV sequence and primer containing oligo dT sequence (Supplementary Table S1).

**Table 1:** Case records of cats from Aoshima Island that showed severe flu-like symptoms during the first epidemic.

Cat	Date of examination	Adult / Juvenile	Breed/Sex	Symptom (Treatment and medications used)	CPE in CRFK cell	RT-PCR	
						FCV	FPV
Ama	2020.11.6	Adult	Mixed-breed/F	Severe flu-like symptom (Clarithromycin, doxycycline, famciclovir, feline interferon)	N.T.	N.T.	N.T.
Dokin	2020.11.6	Adult	Mixed-breed/M	Severe flu-like symptom, oral ulcer (Clarithromycin, doxycycline, famciclovir, feline interferon)	N.T.	N.T.	N.T.
Waru	2020.11.6	Adult	Mixed-breed/F	Severe flu-like symptom, oral ulcer (Clarithromycin, doxycycline, famciclovir, feline interferon)	N.T.	N.T.	N.T.
Char	2020.11.12	Adult	Mixed-breed/M	Severe flu-like symptom, oral ulcer (Clarithromycin, doxycycline, famciclovir, feline interferon)	+	+	−
Kotori	2020.11.12	Adult	Mixed-breed/F	Severe flu-like symptom, hypothermia, oral ulcer (Clarithromycin, doxycycline, famciclovir, feline interferon)	+	+	−
Shiro	2020.11.12	Adult	Mixed-breed/F	Severe flu-like symptom (Clarithromycin, doxycycline, famciclovir, feline interferon)	+	+	−
Scarlet	2020.11.18	Adult	Mixed-breed/M	Severe flu-like symptom, diarrhea (Clarithromycin, doxycycline, famciclovir, feline interferon)	+	+	−
Hoho	2020.12.5	Adult	Mixed-breed/F	Severe common flu-like symptom, anemia, diarrhea, dehydration, large numbers of roundworms, death (Orbifloxacin, selamectin, sarolaner, metronidazole)	+	+	−
John	2020.12.5	Adult	Mixed-breed/M	Severe flu-like symptom, anemia, diarrhea, dehydration (Orbifloxacin, selamectin, sarolaner, metronidazole, clarithromycin, famciclovir, feline interferon, blood transfusions)	+	+	−

Adult cats; 1 year and older.

M; Male, F; Female.

FCV, feline calicivirus; N.T., Not tested because no pharyngeal swab was collected.

+; Positive, −; Negative-.

### 2.6 Neutralization test

Serial dilutions of cat serum were prepared and tested for virus neutralization (final volume: 50 µL/well). Each sample was added at a ratio of 1:2 to the dilution media (DMEM-10FBS), and two-fold serial dilutions were performed in quadruplicate. Equal volumes of dilution medium containing 100 TCID_50_ of any of the FCV isolates were added to each well and incubated at 37 °C for 1 h. Virus-serum mixtures were added to confluent CRFK cells seeded in 96-well plates. The 50% neutralization dose (ND_50_) for 50 µl of cat serum was calculated according to the Behrens–Kärber method (Kärber, 1931).

To obtain serum for cross-neutralization testing, blood samples were collected from a cat on Aoshima Island in March 2023, and pharyngeal swabs and serum were collected from a Scarlet, who recovered and survived after showing severe flu-like symptoms in 2020. The FCV was isolated from the collected swabs (FCV/Aoshima/Scarlet/2023). At this time, pharyngeal swab fluid and serum were collected from one cat (ID: FAS2023-118) on the island, and FCV/Aoshima/FAS2023-118/2023 was isolated from the swab fluid. There is also a record of Scarlet being brought to the veterinary clinic in Shikoku by a volunteer for dental care in late March 2022 and examined; mouth ulcers were also observed during the dental care. At that time, Felivac-L3 (Kyoritsu Seiyaku, Tokyo, Japan), a combination live vaccine against FCV, FHV-1, and FPV, was also administered to Scarlet. Serum collected from a cat vaccinated with the F9 strain-based vaccine was kindly provided by a veterinary hospital in Osaka and used as a control sample for the cross-neutralization test. The cat had a history of three FCV vaccinations: the first with Virbagen CRP (Vibac, Carros, France), and the second and third with TRICAT (MSD Animal Health, Rahway, NJ, USA). Four weeks after the third vaccination, blood was drawn and serum was collected.

## 3 Results

### 3.1 Case history

**Fig. 1:**
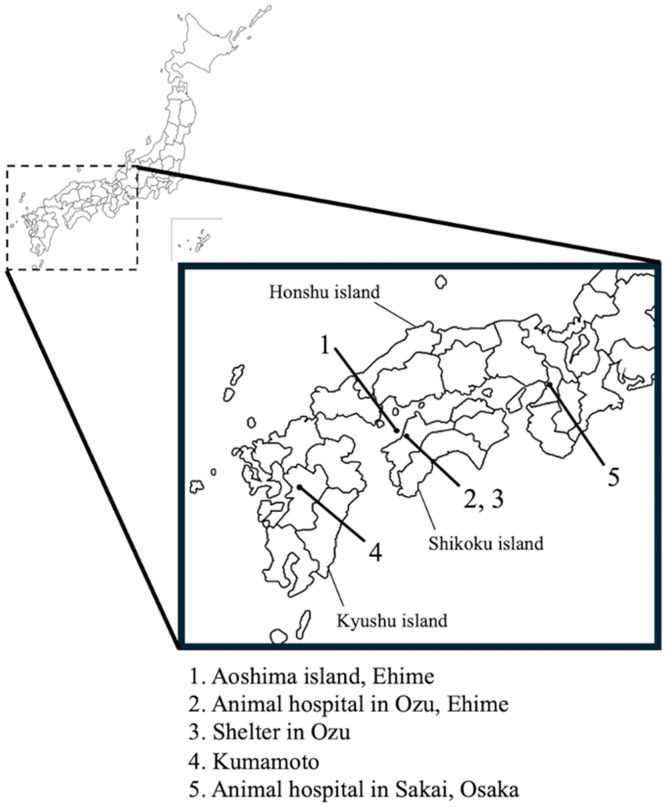
Location of Aoshima Island in Japan (1) and where specimens were collected around the remote island. Nine cats with severe colds in Aoshima Island were hospitalized and treated at a veterinary hospital in Ozu City, Ehime Prefecture (2) and pharyngeal swab samples were collected from six of the hospitalized cats. Pharyngeal swab samples were also collected from cats maintained at a cat shelter in Ozu City (3), local cats kept and managed in Kumamoto Prefecture, without their owners (4), and a cat that visited a veterinary hospital in Sakai city, Osaka Prefecture (5), respectively. Dots indicate the location of specimen collection.

In October 2018, a group of volunteers with a clinical veterinarian conducted trap-neuter-return activities on Aoshima Island and all cats were spayed or neutered. Thus, all cats on Aoshima Island were adults during the emergence of infectious diseases in late October 2020. From late October to early November 2020, island residents and a volunteer group engaged in cat protection activities on Aoshima Island observed that many cats showed severe flu-like symptoms and four cats died. Around this time, three cats, named Ama, Dokin, and Waru, showing severe flu-like symptoms were discovered by a member of the volunteer group (Table 1) and were transported from the remote island to a veterinary hospital in Shikoku for hospitalization and treatment (Fig. 1). A tablet cocktail of antibiotics and anti-herpesviral including clarithromycin, doxycycline, and famciclovir, was administered orally to each cat (Table 1). In addition, feline interferon was also administered. The three cats recovered by November 12 and were discharged and returned to Aoshima Island. Subsequently, a serious flu outbreak was further recognized by volunteers, and photos of animal carcasses taken and documented by volunteers confirmed that at least five cats had died in Aoshima on November 12, 2020. Volunteer groups buried the carcasses on the island. Simultaneously, three cats (Char, Kotori, and Shiro) with severe flu-like symptoms were discovered and delivered to the same veterinary hospital for examination and treatment (Table 1). The hospitalized cats recovered by November 24, 2020, and were brought back to Aoshima Island. A cat, designated as Scarlet, was also found to exhibit severe flu-like symptoms and was taken to the hospital on November 18, 2020, but recovered and returned to the island on December 5, 2020, after receiving the same medication as Char, Kotori, and Shiro had received (Table 1). Two other cats, Hoho and John, were admitted to the veterinary clinic on December 5, 2020, with severe flu-like symptoms. In addition, Hoho exhibits anemia and diarrhea, and its feces contained numerous roundworms. Both cats were treated with a fluoroquinolone antibiotic (orbifloxacin) and anthelmintics for roundworms and protozoa (Table 1). However, their symptoms did not improve within a few days of administration. Hoho died on December 7, two days after admission (Table 1 and Supplementary Table S2). John showed symptoms of anemia, diarrhea, and dehydration in addition to common flu-like symptoms (Table 1 and Supplementary Table S2). After Hoho died, John underwent the similar drug treatment as Char, Kotori, and Shiro had received, with clarithromycin, famciclovir, and feline interferon, but its health condition showed little improvement. As severe anemia did not resolve, blood transfusions were administered. John subsequently went into remission and was discharged from the veterinary hospital on December 23, 2020. However, the cat was deemed to need bed rest and cared for at the volunteer's home. By May 30, 2021, John had fully recovered from the symptoms and returned to Aoshima Island.

### 3.2 Virus isolation and virus genome detection

**Fig. 2:**
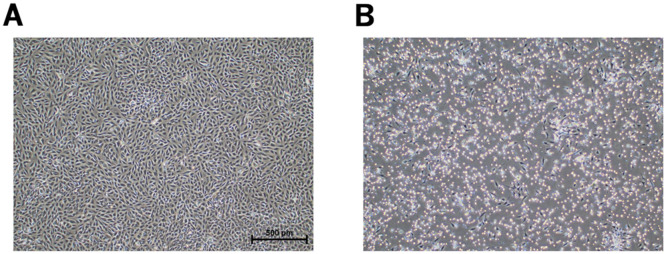
CPE observed on the CRFK cells inoculated with the pharyngeal swab fluid collected from the six cats showing severe flu-like symptoms. (A) The cell morphology of the mock-infected CRFK cells is shown. (B) A representative image showing the CPE. Cell circularization and detachment was observed in the CRFK cells 12 h after inoculation with the supernatant of swab suspension from a cat named Hoho. Phase-contrast micrographs were shown at 40 × magnification. CPE, cytopathic effect.

To investigate the etiological virus in cats with severe flu-like symptoms, we attempted to isolate the virus from pharyngeal swab fluid collected from cats admitted to a veterinary hospital (Table 1). Because swab samples were obtained from only 6 of the 9 hospitalized cats (Char, Kotori, Shiro, Scarlet, Hoho, and John) (Table 1), and supernatants from the suspensions were inoculated into CRFK, Fcwf-4, or Vero/DogSLAM cells. In CRFK and Fcwf-4 cells, CPE, which is characterized by cell circularization, was observed as early as 10–24 h after inoculation in all samples (Fig. 2). In contrast, CPE was not detected in Vero/DogSLAM cells (data not shown).

Next, we attempted to detect viral RNAs using a viral species-specific RT-PCR method to identify the species of the isolated viruses. FCV infection was strongly suspected based on the CPE pattern observed during the early stages of infection. On the other hand, parvovirus infection was also considered, as the cat showed suspected systemic symptoms. PCR was performed using consensus primers targeting FCV or FPV. The FCV gene was detected in all samples, whereas the PCR targeting FPV was negative (Table 1).

### 3.3 Search for pathogens by NGS

A pooled sample (pool#1) was prepared by mixing the RNA samples extracted from the pharyngeal swabs of the six cats with severe flu-like symptoms (Char, Kotori, Shiro, Scarlet, Hoho, and John). For some diseased animals (Dokin, Waru, Char, Kotori, and Shiro), a small amount of serum was collected during hospitalization and mixed in equal amounts to create a serum pool solution, following RNA extraction (pool#2). Pooled RNA samples derived from pharyngeal swabs and sera (pool#1 and pool#2) were further analyzed using deep sequencing. Many FCV gene fragments were detected in the pharyngeal swab pooled sample (pool#1). Of the 83,353,512 leads, 1412 (less than 0.01%) mapped to the FCV sequence. In addition, de novo assembly was performed on all reads except those mapped to FCV, and BLAST searches of the resulting sequences yielded 73 hits for the feline genome sequence. No gene fragments from other viral species were identified (Table 2). In contrast, no viral gene fragments were identified as significant sequences with sufficient coverage in the pool of serum-derived RNA (pool#2). Instead, hemoplasma gene fragments were detected (Table 2). After assembling over 46,000 reads, a 2.4 kbp long fragment of the *23S* and *5S* rRNA genes of the hemoplasma was reconstructed. A BLAST search of the constructed sequence revealed 99% identity to gene sequences of *Candidatus* Mycoplasma haemominutum (Table 2).

**Table 2:** Pathogen gene fragments identified by NGS.

Pooled sample	Specimen in pool (individual name)	Detected gene fragments
Pharyngeal swab suspension (pool#1)	Char	FCV
	Kotori	
	Shiro	
	Scarlet	
	Hoho	
	John	
Serum (pool#2)	Dokin	*Candidatus M. haemominutum*
	Waru	
	Char	
	Kotori	
	Shiro	

FCV, feline calicivirus.

### 3.4 Epidemiological survey of FCV in Aoshima Island and western parts in Japan after the initial epidemic of severe flu-like disease

On December 27, 2020, after the outbreak of infectious diseases in cats had ended, 22 cats were captured in Aoshima for pharyngeal swab collection (Supplementary Table S3). For comparison with other FCVs distributed to cats in the area around Aoshima Island, pharyngeal swab fluid was collected from cats housed in a shelter in Ozu, Ehime Prefecture; a cat that visited a veterinary clinic in Sakai, Osaka Prefecture; and a cat maintained or cared for by a local community in the Kumamoto area (Fig. 1 and Supplementary Table S4). After preparing a suspension for each pharyngeal swab specimen, CRFK cells were inoculated for virus isolation. CPE characterized by cell rounding was observed in 18 of 22 samples from cats in Aoshima Island, 5 of 10 samples from shelter cats in Ozu, Ehime, 3 of 14 samples from a clinic in Osaka, and 3 of 20 samples from cats kept by the local community in Kumamoto (Supplementary Table S3 and Supplementary Table S4). As FCV infections were suspected based on the observed CPEs, RT-PCR was conducted. FCV RNAs were detected in almost all pharyngeal swabs from the individual cats from which the virus was isolated (Supplementary Tables S2 and S3). For the 22 samples collected during the epidemiological survey in Aoshima, RT-PCR targeting FPV was also performed, and all samples tested negative for FPV (Supplementary Table S3).

### 3.5 Determination of sequencing of FCV isolates and molecular phylogenetic analysis of their sequences

**Fig. 3:**
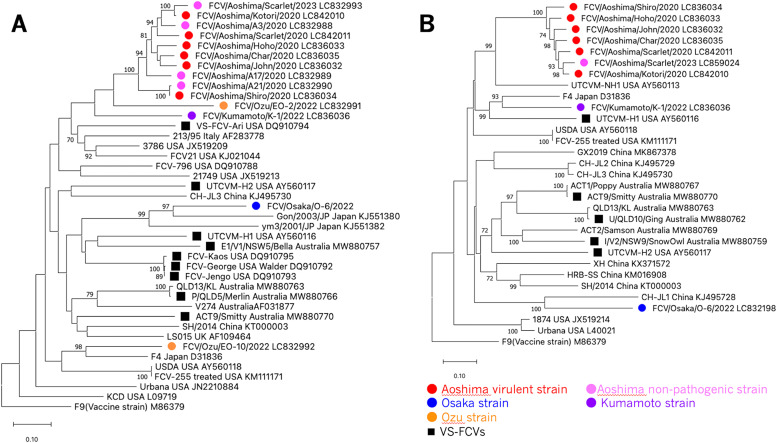
Phylogenies of the FCVs isolated in Aoshima Island and western parts of Japan. Phylogenetic tree based on (A) complete *VP1* gene or (B) nucleotide sequences of complete full-genome of FCVs was reconstructed with Maximum Likelihood method using MEGA11 (Stecher et al., 2020; Tamura et al., 2021). Percentage of replicate trees in which the associated taxa clustered in the bootstrap test (1000 replicates) is calculated and values above 70% are shown next to the branches. The tree is drawn to scale, with branch lengths measured in the number of substitutions per site. The tree was rooted to a vaccine strain, F9. Names of the FCV strains used for comparison and their GenBank accession numbers are shown in each element or leaf. Type of each virus strain is also indicated by a colored circle or black square as shown in explanatory note.

Using primers based on the sequence of the F9 vaccine strain (Supplementary Table S1), the full-length sequence of the *VP1* gene was determined using direct sequencing for atypical virus strains isolated in Aoshima Island (Aoshima virulent strains), from the six cats with severe flu-like symptoms (Char, Kotori, Shiro, Scarlet, Hoho, and John). Of the six animals, Scarlet was found to live on Aoshima Island in 2023 after its symptoms recovered, and FCV (FCV/Aoshima/Scarlet/2023) was again isolated from pharyngeal swabs. Subsequently, the *VP1* gene sequence was determined. Moreover, three canonical FCV strains, FCV/Aoshima/A3/2020, FCV/Aoshima/A17/2020, and FCV/Aoshima/A21/2020, isolated from cats in Aoshima during an epidemiological survey conducted in late December 2020 (after the end of the epidemic), were randomly selected (Supplementary Table S3), and the full-length *VP1* gene sequence was determined in the same manner. In addition, to compare the phylogenetic relationships with the Aoshima isolates, four representative strains from western Japan, FCV/Ozu/EO-2/2022, FCV/Ozu/EO-10/2022, FCV/Osaka/O-6/2022, and FCV/Kumamoto/K-1/2022, which were newly isolated in the present study (Supplementary Table S4), were selected, and the full-length sequence of the *VP1* gene was also determined. A molecular phylogenetic tree was constructed based on the complete VP1 genome sequences of the 14 newly sequenced strains, along with the known FCV strains. The resultant tree showed that the virulent Aoshima strains clustered in the same phylogenetic group as the Aoshima non-pathogenic strains. Moreover, the Aoshima virulent and non-pathogenic strains formed an independent phylogenetic group (Aoshima isolate strains) distinct from known FCVs, including the VS-FCV strains (Fig. 3A).

Next, the full-length sequences of the FCV isolates were further determined using NGS and/or direct sequencing with primer walking, for all the Aoshima virulent strains, two isolates from western Japan, FCV/Osaka/O-6/2022 and FCV/Kumamoto/K-1/2022 (Supplementary Table S4), and the 2023 isolate from Scarlet recovering from severe flu-like symptoms (FCV/Aoshima/Scarlet/2023). A phylogenetic tree was constructed from the determined full-length and known FCV sequences, confirming that the virulent Aoshima strains and the FCV/Aoshima/Scarlet/2023 formed an independent phylogenetic group (Fig. 3B).

### 3.6 Antigenicity of FCV

To compare the antigenicity among the FCV isolates, cross-neutralization tests were performed using the FCV isolates and sera collected from cats from which the virus was isolated. The results showed that the Osaka isolate had low cross-reactivity with the virulent Aoshima and vaccine strains (Table 3). In a neutralization test using serum from a cat infected with the Osaka strain, the neutralizing antibody titer was 48,710 when the Osaka strain was used, whereas the titers were as low as 1522 and 5120 when the Aoshima virulent and vaccine strains were used, respectively. Moreover, antibody titers in neutralization tests using serum from vaccinated cats were as low as 269 when the Osaka strain was used, compared to 57,926 when attacked by the vaccine strain (Table 3). In contrast, moderate reactivity was observed for the Aoshima virulent strain (24,355) and a strain from the Aoshima non-pathogenic strain, FCV/Aoshima/FAS2023-118/2023 (10,240) (Table 3). Thus, the vaccine strain shared poor cross-reactivity with the Osaka strain but a certain degree of cross-reactivity with the Aoshima isolates. Furthermore, serum from cats infected with the virulent Aoshima strain showed little cross-reactivity with the Osaka strain when collected again after recovery from the disease in 2023 (1810 vs. 1076) (Table 3). In addition, the serum collected in 2023 from cats in Aoshima with no history of severe symptomatic illness or vaccination (serum against FCV/Aoshima/FAS2023-118/2023) showed a certain degree of cross-reactivity, with antibody titers of 10,240 and 10,240 in neutralization tests with the Osaka and vaccine strains, respectively (Table 3).

**Table 3:** Virus neutralization titers of FCV isolates from symptomatic cats in the animal hospital and cats caught in epidemiological surveys.

	Serum				
Virus	FCV/Osaka/O-13/2022	FCV/Aoshima/Scarlet/2020	FCV/Aoshima/Scarlet/2023	FCV/Aoshima/FAS2023-118/2023	F9
FCV/Osaka/O-13/2022	48,710	1810	1076	10,240	269
FCV/Aoshima/Scarlet/2020	1522	40,960	24,355	8611	24,355
FCV/Aoshima/Scarlet/2023	905	28,963	48,710	4305	10,240
FCV/Aoshima/FAS2023–118/2023	320	N.T.	N.T.	20,480	10,240
Vaccine (F9)	5120	N.T.	N.T.	10,240	57,926

FCV, feline calicivirus; N.T., Not Tested.

## 4 Discussion

Although Aoshima Island is known to contain approximately 100 wild cats, the number of humans living on the island has decreased, with only a few residents. A volunteer group that cares for feral cats regularly visits the island to monitor their health. Microchip installation for individual identification began in November 2021, and cats that had not yet been equipped were underwent microchip installation installed in every survey. It is thought that all cats have been identified to date. By October 2024, 107 cats had been chipped, although the exact number of cats that were alive was unknown. Through these health observations, it was recognized that infectious diseases with flu-like symptoms are prevalent in cats every winter. However, during the winter season of 2020 (late October 2020 to December 5, 2020), many cats showed marked flu-like symptoms, and nine cats with particularly severe symptoms were transported by a volunteer group to a veterinary hospital located in Shikoku for hospitalization and treatment (Table 1 and Fig. 1). The symptoms did not improve after administering a broad-spectrum antibiotic mixture to the hospitalized cat, therefore, a viral infection epidemic was suspected.

To determine the etiology of the infection, viruses were isolated from pharyngeal swabs collected from cats treated at a veterinary hospital. As a result, CPE characterized by cell rounding was observed in cultured cells derived from feline renal or fetal tissue within 1 d after inoculation (Fig. 2). The observation of CPE in CRFK or Fcwf-4 cells raised the possibility of infection with FCV or FHV-1, which cause upper respiratory tract infection in cats. Since FHV-1 infects CRFK cells and fusible CPE is observed in the late stages of infection (usually after day 2) (Henzel et al., 2012), it is unlikely that infection by FHV-1 or reactivation of high levels of latent infected FHV-1 is occurring. Furthermore, FCV RT-PCR detected the viral gene, indicating that the six hospitalized and treated cats were infected with FCV (Table 1). FCV typically causes no severe symptoms and subclinical infections in cats. Assuming infection by viral pathogens other than FCV, we attempted gene detection using RT-PCR targeting FPV; however, the FPV RNA was not detected (Table 1). In 2020, COVID-19 emerged and caused a worldwide pandemic. Thus, the transmission of novel coronaviruses to cats has also been assumed, as cats have been reported to be susceptible to the novel coronavirus (Chiba et al., 2021). However, we did not observe CPE due to coronavirus infection in Fcwf-4 cells susceptible to novel coronaviruses or feline coronaviruses (data not shown), suggesting that the cats were not infected with coronaviruses, including novel coronaviruses. Furthermore, the results of virus isolation using Vero/DogSLAM cells indicated that it is unlikely that the diseased animal was infected with other pathogens that could grow in Vero cells (data not shown). In addition, RNA was extracted from pharyngeal swab suspensions or pooled serum samples to search for pathogen genes using NGS, which identified numerous FCV and ribosomal genes in the hemoplasma (Table 2). Collectively, it is unlikely that a viral pathogen other than FCV infected hospitalized cats.

Although feline hemoplasma has been reported to cause hemolytic anemia in cats (Inokuma, 2005 ), it has also been detected in healthy cats. Epidemiological studies on cats visiting veterinary clinics in Japan have reported high carriage rates of approximately 40% (Inokuma et al., 2004; Tanahara et al., 2010). Three major Hemoplasma species are known to infect cats: *Mycoplasma haemofelis, Candidatus* M. haemominutum, and *Candidatus* M. turicensis. *M. haemofelis* is highly virulent, causing acute infection with hemolytic anemia, fever, and anorexia, sometimes leading to fatalities. In contrast, the other two strains are relatively less virulent and are thought to cause few clinical symptoms when infected alone, although cases of co-infection with the feline leukemia virus causing anemia have been reported (George et al., 2002). In this study, NGS revealed genomic fragments of *Candidatus* M. haemominutum in pooled serum samples. In general, hemoplasmas are present in blood cells but not in serum samples. Thus, it is assumed that a substantial amount of the hemoplasma genome was present in the blood of any of the cats, Dokin, Waru, Char, Kotori, and Shiro, to which pooled serum was provided. Unfortunately, no remaining serum samples were obtained from any of the five cats. In addition, because blood cells and whole blood were not collected and stored, it was not possible to examine the hemoplasma infection status of the five animals in detail at the time of admission. Importantly, despite the possible presence of large amounts of haemoplasma in the blood, there were no symptoms or test results indicative of anemia in these five cats. Therefore, it is unlikely that hemoplasma contributed to the illness in the five cats that showed anemia. Furthermore, Char, Kotori, and Scarlet were found on Aoshima Island in March 2023 after recovering from their symptoms. Whole blood samples were taken during health observations and observed to be in good health. DNA was extracted from blood samples, and hemoplasma gene detection was performed using primers targeting the hemoplasma *16* *s* rRNA gene. However, no hemoplasma gene was detected in samples from Char and Kotori, whereas amplification of the hemoplasma gene was detected in samples from Scarlet (data not shown). These findings suggest that *Candidatus* M. haemominutum infection is not highly pathogenic in cats.

On the other hand, no blood or serum samples were left for Hoho and John, who showed anemia, and there were no findings indicating a contribution of *Candidatus* M. haemominutum in the current study. Parasitic eggs of the feline roundworm, *Toxocara cati*, were detected in the deceased Hoho. In general, *T. cati* does not cause anemia or fatalities in cats; however, gastrointestinal symptoms have been observed. John also reported symptoms indicative of anemia, suggesting that anemia is a symptom of FCV infection. Symptoms of jaundice and anemia have been reported in previous outbreaks of VS-FCV, and the possibility that the virulent Aoshima strain isolated in this study was as virulent as the VS-FCV strain cannot be ruled out. However, the typical symptoms of VS-FCV infection, such as edema and ulcers on the face and body surfaces, except for the oral cavity, were not observed in Hoho and John when they were hospitalized for acute infection (Radford et al., 2007). Similarly, no abnormalities were observed on the body surfaces of Shiro, Char, Kotori, Dokin, and Waru, and the FCV genomes were not detected in the pooled sera collected and prepared as described above. We were unable to confirm viremia, which is often observed in VS-FCV-infected cats, owing to the limited number of specimens collected in this study during the current outbreak. These results indicate that the virulent Aoshima strain isolated in this study was not a typical VS-FCV strain. However, symptomatic FCV infection is not usually observed in adult cats, suggesting that the virulent Aoshima strain is highly pathogenic. A highly virulent strain of FCV, FCV-255, has been reported to cause pneumonia in feral cats, although its infectivity of FCV strain in adult cats is unknown (Gillespie and Scott, 1973). Similar to FCV-255, the virulent Aoshima strain may cause severe respiratory infections.

Phylogenetic tree analysis based on the full-length sequence of the VP1 region showed that the virulent and Aoshima non-pathogenic strains detected in this study were located within the same phylogenetic group (Aoshima isolates) and formed an independent phylogenetic group (Fig. 3A). The isolates from Aoshima Island also showed a close phylogenetic relationship with the western Japanese isolates that were first isolated in this study. The Aoshima strains were most closely related to the Ozu strain, which was also isolated in Ehime Prefecture, and showed a close phylogenetic relationship with a strain isolated in Kumamoto Prefecture, which is relatively geographically close (Fig. 1, Fig. 3A). In contrast, the Osaka isolate was more closely related to the Tokyo isolates, whose sequences have been registered in the database as direct submissions (Gon/2003/JP; accession number: KJ551380, ym3/2001/JP; accession number: KJ551382), and showed a distant phylogenetic relationship with the Ehime isolates. The VS-FCV strains or suspected strains already reported abroad show a close phylogenetic relationship with the canonical FCV strains isolated in the surrounding area during the same period (Bordicchia et al., 2021), Similarly, in this study, the virulent Aoshima strain showed high homology with the Aoshima non-pathogenic strain (Fig. 3A). Since the pathogenic and nonpathogenic strains were located in the same phylogenetic group, even when compared by full-length genes (Fig. 3B), the number of genetic variants involving amino acid mutations between the two strains is expected to be small. However, a comparison of the full-length sequences of strains isolated from cats showing flu-like symptoms and those isolated again from the same cats three years after recovery revealed many nucleotide sequence variations (FCV/Aoshima/Scarlet/2020 vs FCV/Aoshima/Scarlet/2023) (Supplementary Table S5). Non-synonymous substitutions are not limited to the VP1 region, but mutations were found into genes throughout the entire length of the viral genome, suggesting that the genetic diversity of FCV is extremely high originally, considering the topology of the phylogenetic tree. Furthermore, the virulent Aoshima strain was not closely related to FCV-255, a pneumotropic strain. These facts suggest that VS-FCV strains from other regions were unlikely to have been introduced to Aoshima Island. This is consistent with the fact that the only access to Aoshima Island from other regions is a small ferry from the main island of Shikoku, with almost no opportunity for cats from distant areas, such as Honshu, the largest main island of Japan, to enter the remote island (Fig. 1).

The results of the cross-neutralization test showed that the Osaka strain had low cross-reactivity with the vaccine strain, whereas the Osaka and Aoshima isolates showed some degree of cross-reactivity (Table 3). This may indicate that cats in urban areas are vaccinated with core vaccines, including the FCV vaccine, and viruses with high cross-reactivity with the vaccine strains are less likely to spread among urban cats because of the vaccine's preventive effects against infections. In contrast, cats on Aoshima Island have not been core vaccinated; therefore, the Aoshima isolate is likely to show some degree of high cross-reactivity with the vaccine strain. Furthermore, a high degree of cross-reactivity was observed between pathogenic strains derived from hospitalized cats and other Aoshima non-pathogenic strains (Table 3). This is consistent with the fact that the genetic sequences of the Aoshima strains are closely related. Therefore, it is inferred that the outbreak of a new FCV with completely different antigenic properties did not cause this severe outbreak; rather, the virus was maintained on a small remote island, which has been chronically infecting feral cats, mutated, and a virulent strain emerged.

Brunet et al. reported that comparing amino acid variations in the E region of the hypervariable region of VP1 among FCV strains, performed principal component analysis (multiple correspondence analysis), and identified the factors that most correlated with the virulence of FCV strains (VS-FCV vs. classical FCV) (Brunet et al., 2019). The E region is located at amino acid site 426–521 of VP1 and is also involved in interaction with the FCV entry receptor, feline JAM-A. The analysis identified that differences in the features or chemistry of the seven amino acid sites in the E region (positions 438, 440, 448, 452, 455, 465, and 492) were highly correlated with distinguishing VS-FCV strains from classical strains. As shown in Supplementary Table S6 and Supplementary Fig. S1, the amino acid pattern of the seven amino acid sites of the Aoshima virulent strain showed properties intermediate between those of the VS-FCV type and the canonical strain type proposed by Brunet et al. (Brunet et al., 2019). On the other hand, two of the three new classical strains isolated in western Japan in this study were of Brunet's VS-FCV type. Thus, the result shows that the amino acid pattern of the classical strain type proposed by Brunet et al. was not maintained in any of the canonical strains (Supplementary Table S6). Notably, the 7-site amino acid pattern of the Aoshima virulent strain shows intermediate properties that do not fit either pattern.

FCV infections have been reported in cats worldwide. Although the risk of severe disease in kittens and immunocompromised adult cats is known, this is not generally known in healthy adult cats (Green, 2013; Radford et al., 2007). As already mentioned, all cats on Aoshima Island were adults, suggesting that the Aoshima virulent strain found in this study, which caused severe flu-like symptoms, is a highly virulent strain that is different from the known canonical strain of FCV. Including animals for which specimens could not be collected, our records indicated that 10 individuals died, which is a very high mortality rate, considering the population size of approximately 100 animals. Further studies are needed to clarify the pathogenicity of the Aoshima isolate in cats, including confirmation of the reproducibility of symptoms by experimental infection. It is suspected that most cats on the island were persistently and chronically infected with FCV, as FCVs were isolated from most individuals that did not show disease in surveys after the end of the epidemic (Supplementary Table S3). Although symptomatic infections with typical FCV strains often occur in shelters and other large-scale breeding facilities, the environment of a very small island with approximately 100 stray cats in the present study may have served as a natural shelter.

## Data availability

The nucleotide sequence data reported herein have been deposited in the DNA Data Bank of Japan (DDBJ) under accession numbers LC832198, LC832988-LC832994, LC836032-LC836036, LC842010-LC842011, and LC859024. All other data will be provided upon request.

## Ethical approval

All animal experiments were reviewed and approved by the Animal Experiments Committee of Okayama University of Science and were carried out in accordance with the Regulations for Animal Experiments of Okayama University of Science (#2021–032).

## CRediT authorship contribution statement

**Yuki Nishisaka:** Writing – review & editing, Writing – original draft, Visualization, Investigation, Formal analysis. **Hikaru Fujii:** Writing – review & editing, Visualization, Investigation, Formal analysis, Conceptualization. **Fumiko Ono:** Writing – review & editing, Project administration, Investigation, Funding acquisition, Conceptualization. **Sho Kadekaru:** Writing – review & editing, Investigation. **Hiroyuki Kogiku:** Writing – review & editing, Investigation. **Yumi Une:** Writing – review & editing, Investigation. **Shione Takeguchi:** Writing – review & editing, Investigation. **Naomi Ohta:** Writing – review & editing, Formal analysis. **Masumi Eto:** Writing – review & editing, Supervision. **Chiharu Takeuchi:** Writing – review & editing, Investigation. **Seigou Takeuchi:** Writing – review & editing, Investigation. **Tetsuko Miki:** Writing – review & editing, Investigation. **Akihiko Tokuda:** Writing – review & editing, Investigation. **Keiko Ookawa:** Writing – review & editing, Investigation. **Yukinobu Tohya:** Writing – review & editing. **Keita Ishijima:** Writing – review & editing, Formal analysis. **Akiko Okutani:** Writing – review & editing, Formal analysis. **Ken Maeda:** Writing – review & editing, Project administration. **Shumpei Watanabe:** Writing – review & editing, Writing – original draft, Visualization, Supervision, Project administration, Investigation, Funding acquisition, Formal analysis, Conceptualization. **Shigeru Morikawa:** Writing – review & editing, Supervision, Project administration, Funding acquisition, Conceptualization.

## Declaration of competing interest

The authors declare that they have no known competing financial interests or personal relationships that could have appeared to influence the work reported in this paper.

## Data Availability

Data will be made available on request.

## Appendix Supplementary materials

Image, image 1

Image, application 2
