# Supplementary figures and images for: Molecular characterization of feline caliciviruses isolated from several adult cats with atypical infection showing severe flu-like symptoms on a remote island in Ehime, Japan
Source: Virus Res. 2025 Jan 30;353:199535. doi: 10.1016/j.virusres.2025.199535 (PMC11830335; doi:10.1016/j.virusres.2025.199535)

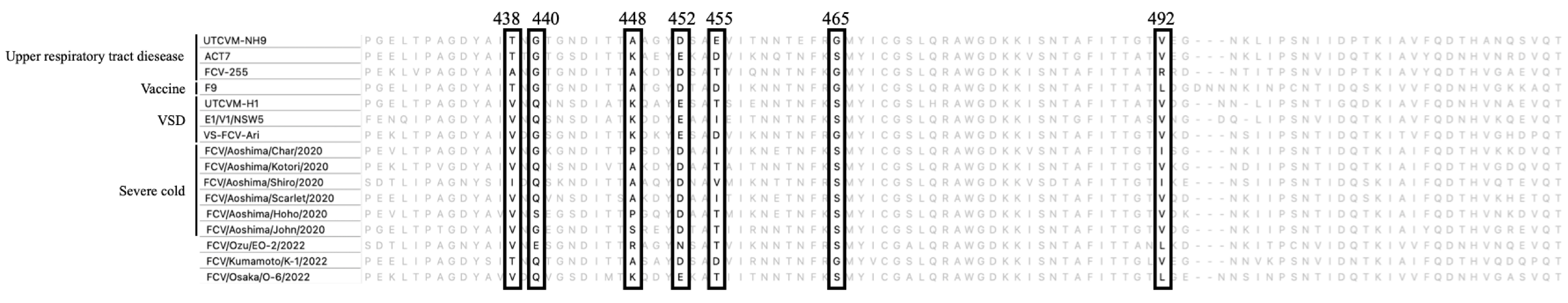

Supplement: Supplementary file 1 [file mmc1.jpg]
