# Supplementary material for: Molecular characterization of feline caliciviruses isolated from several adult cats with atypical infection showing severe flu-like symptoms on a remote island in Ehime, Japan
Source: Virus Res. 2025 Jan 30;353:199535. doi: 10.1016/j.virusres.2025.199535 (PMC11830335; doi:10.1016/j.virusres.2025.199535)
Supplement: Supplementary file 2 [file mmc2.docx]

**Supplementary Materials**

**Supplementary Fig. S1.**

Alignment of the hypervariable E-region of VP1. The seven positions, proposed by Brunet et al., which are important for distinguishing VS-FCV strains from classical strains, are indicated by vertical squares with the number of VP1 amino acid sites.

**Supplementary Table S1.**

Primers used to determine the complete genome of the FCV strains.

| **Primers** | **Sequences（5ʹ-3ʹ）** | **Position in the full-length gene sequence of the F9 strain** | **Reference** |
| --- | --- | --- | --- |
| Cali1 | AACCTGCGCTAACGTGCTTA | 5322-5341 | (Marsilio et al., 2005) |
| Cali2 | CAGTGACAATACACCCAGAAG | 6227-6247 |  |
| Cali4 | ACACCAGAGCCAGAGATAGA | 5971-5990 |  |
| Mao primer-f | TTGAGCATGTGCTCAACCTG | 5308-5328 | (Mao et al., 2022) |
| Mao primer-r | ATTTGRTTTGTATGAGTAAGGG | 7562-7583 |  |
| F9-1f | GTAAAAGAAATTTGAGACAATGTCTCAAACTCTGAGC | 1-37 | (Guo et al., 2018) |
| F9-1r | CTCATGCAAGATCTTAGCCAATTC | 1292-1315 | − |
| F9-2f | ATCATGCAATCATACAACCCAAT | 1343-1365 |  |
| F9-2r | CCNADDGCCCAGATDCGHGCHCC | 2666-2688 |  |
| F9-3f | TGGTGGTGCDCGHATCTGGGC | 2662-2682 |  |
| F9-3r | CCYTGNGTDGCCAATTTCCATTTTG | 4182-4206 |  |
| F9-4f | TCHKCAAAATGGAAATTGGC | 4178-4197 |  |
| FCV-1r | TCATCAGGGCATAACTCGTC | 308-327 | − |
| FCV-2f | AGGCTTGCTATTGTAAATCG | 612-631 |  |
| FCV-3r | CTCATGCAAGATCTTAGCCAATTC | 1292-1315 |  |
| FCV-4f | ATCATGCAATCATAFCAACCCAAT | 1345-1367 |  |
| FCV-5f | GCGTGGAGCAGAGTGTTGGTG | 1917-1937 |  |
| FCV-6r | GCGGTGCACCCTACAGAGTTAG | 2207-2228 |  |
| FCV-7f | TGGTGGTGCDCGHATCTGGGC | 2664-2684 |  |
| FCV-8r | CCNADDGCCCAGATDCGHGCHCC | 2666-2688 |  |
| FCV-9f | GTGGCACATGTTGTTAAGGGTG | 3346-3367 |  |
| FCV-10r | GTTGTGTATGGTTTGGGCTTC | 3514-3534 |  |
| FCV-11r | CCYTGNGTDGCCAATTTCCATTTTG | 4182-4206 |  |
| FCV-12f | TCHKCAAAATGGAAATTGGC | 4183-4202 |  |
| FCV-13f | GGACTACTTGACCGTAGTTC | 4970-4989 |  |
| FCV-14r | CCACGAGATGTTGGGTCAATTG | 5054-5076 |  |
| FCV-15f | GGAGTCATCGCTGAACCCAGTACC | 5734-5757 |  |
| FCV-16r | CTTTCCTTGTGTTTCAGTTGTGCTCC | 5846-5871 |  |
| FCV-17f | CTATATCTGGGTCTGGTGTCTTCG | 5972-5995 |  |
| FCV-18f | GAATGGATCAAAGCTTGGAACTGC | 6507-6530 |  |
| FCV-19r | CTGAATTCCCATGTAGGAGGCGG | 7252-7275 |  |
| FCV-20f | CCTCCTACATGGGAATTCAGTTG | 7256-7278 |  |
| FCV-21f | GGCTGCACTTGGACAACAACGAGAGC | 7400-7425 |  |
| 3siteAd | CTGATCTAGAGGTACCGGATCC | − | − |
| 3siteAd 20T | CTGATCTAGAGGTACCGGATCCTTTTTTTTTTTTTTTTTTTT | − |  |

FCV, feline calicivirus

**Supplementary Table S2.**

Results of complete blood cell count and serum chemistry analysis in cats with anemia and severe flu-like symptoms.

| **Date** | **Parameter** | **Hoho** | **John** | **Reference interval** |
| --- | --- | --- | --- | --- |
| 2020.12.5 | Complete blood count |  |  |  |
|  | RBC (×10^6^/µL) | 4.34 | 7.59 | 5.0–10.0 |
|  | HCT (%) | 22.2 | 27.8 | 24.0–45.0 |
|  | HGB (g/dL) | 6.9 | 9.9 | 8.0–15.0 |
|  | MCV (fL) | 51.2 | 36.6 | 39.0–55.0 |
|  | MCH (pg) | 15.9 | 13 | 12.5–17.5 |
|  | MCHC (g/dL) | 31.1 | 35.6 | 31.0–35.0 |
|  | RDW (%) | 22.3 | 25.7 | 15.0–27.0 |
|  | Reticulocyte (%) | 1 | 0.9 |  |
|  | Reticulocyte (×10^3^/µL) | 42.5 | 70.6 | 3.0–50.0 |
|  | WBC (×10^3^/µL) | 16.99 | 28.32 | 5.5–19.5 |
|  | Segmented neutrophils (×10^3^/µL) | 11.88 | 25.94 | 2.5–12.5 |
|  | Lymphocytes (×10^3^/µL) | 3.57 | 2.03 | 1.5–7.5 |
|  | Monocytes (×10^3^/µL) | 1.45 | 0.34 | 0–0.85 |
|  | Eosinophils (×10^3^/µL) | 0.07 | 0 | 0–1.5 |
|  | Basophils (×10^3^/µL) | 0.02 | 0.01 | 0.01–0.26 |
|  | Platelets (×10^3^/µL) | 331 | 203 | 300–800 |
| 2020.12.6 | Serum chemistry |  |  |  |
|  | GLU (mg/dL) | 29 | N.T. | 73–134 |
|  | CREA (mg/dL) | 0.4 | N.T. | 0.8–1.8 |
|  | BUN (mg/dL) | 40 | N.T. | 20–30 |
|  | BUN/CREA | 101 | N.T. |  |
|  | PHOS (mg/dL) | 6.2 | N.T. | 4.5–8.1 |
|  | CA (mg/dL) | 8.6 | N.T. | 6.2–10.2 |
|  | TP (g/dL) | 8.8 | N.T. | 5.4–7.8 |
|  | ALB (g/dL) | 24 | N.T. | 2.1–3.3 |
|  | GLOB (g/dL) | 6.4 | N.T. | 2.6–5.1 |
|  | ALB/GLOB | 0.4 | N.T. |  |
|  | ALT (U/L) | 71 | N.T. | 6–83 |
|  | ALKP (U/L) | <10 | N.T. | 14–111 |
|  | GGT (U/L) | 0 | N.T. | 0–4 |
|  | Tbil (mg/dL) | 0.2 | N.T. | 0.15–0.50 |
|  | CHOL (mg/dL) | 54 | N.T. | 95–130 |
|  | Na (mEq/L) | 153 | N.T. | 147–156 |
|  | K (mEq/L) | 4.5 | N.T. | 4.0–4.5 |
|  | Na/K | 34 | N.T. |  |
|  | Cl (mEq/L) | 113 | N.T. | 117–123 |

RBC, red blood cell; HCT, hematocrit; HGB, hemoglobin; MCV, mean corpuscular volume; MCH, mean corpuscular hemoglobin ; MCHC, mean corpuscular hemoglobin concentration ; RDW, red blood cell distribution width ; WBC, total white blood cell count; GLU, glucose; CREA, creatinine; BUN, blood urea nitrogen; PHOS, phosphorus; CA, calcium; TP, total protein; ALB, albumin; GLOB, globulin; ALT, alanine aminotransferase; ALKP, alkaline phosphatase; GGT, gamma glutamyl transferase; TBil, total bilirubin; CHOL, cholesterol; Na, sodium, K, Potassium; Cl, chloride; N.T., Not Tested.

**Supplementary Table S3.**

Health records collected during an epidemiological study in Aoshima Island after the epidemic on December 27, 2020.

| **Cat ID** | **Adult / Juvenile** | **Breed/Sex** | **Symptom** | **CPE** | **RT-PCR** | |
| --- | --- | --- | --- | --- | --- | --- |
|  |  |  |  |  | **FCV** | **FPV** |
| A1 | Adult | Mixed-breed/M | Gingivitis、ocular discharge, nasal discharge | + | + | − |
| A2 | Adult | Mixed-breed/M | Oral mucosal pallor, nasal discharge | − | − | − |
| A3 | Adult | Mixed-breed/M | Ocular discharge (right eyeball loss, left eye cloudy ) | + | + | − |
| A5 | Adult | Mixed-breed/F | Ocular discharge | − | − | − |
| A6 | Adult | Mixed-breed/F | － | + | + | − |
| A7 | Adult | Mixed-breed/M | Nasal discharge | − | − | − |
| A8 | Adult | Mixed-breed/M | Ocular discharge, nasal discharge | + | − | − |
| A9 | Adult | Mixed-breed/F | － | + | − | − |
| A10 | Adult | Mixed-breed/M | Gingivitis | + | + | − |
| A11 | Adult | Mixed-breed/M | Gingivitis, ocular discharge, nasal discharge | + | + | − |
| A13 | Adult | Mixed-breed/F | － | + | + | − |
| A14 | Adult | Mixed-breed/M | Gingivitis, ocular discharge, nasal discharge | + | + | − |
| A15 | Adult | Mixed-breed/F | Gingivitis | + | + | − |
| A16 | Adult | Mixed-breed/F | Gingivitis | + | + | − |
| A17 | Adult | Mixed-breed/M | － | + | + | − |
| A18 | Adult | Mixed-breed/F | Gingivitis | − | − | − |
| A19 | Adult | Mixed-breed/F | Gingivitis, ocular discharge, nasal discharge | + | + | − |
| A20 | Adult | Mixed-breed/M | Ocular discharge, nasal discharge | + | − | − |
| A21 | Adult | Mixed-breed/M | Ocular discharge, nasal discharge | + | + | − |
| A22 | Adult | Mixed-breed/M | Ocular discharge, nasal discharge | + | + | − |
| A23 | Adult | Mixed-breed/M | Nasal discharge | + | + | − |
| A24 | Adult | Mixed-breed/F | Ocular discharge, nasal discharge | + | + | − |

The virus was detected in feline pharyngeal swab samples.

Adult cats; 1 year and older

M, Male; F, Female; CPE, cytopathic effect; FCV, feline calicivirus; FPV, feline parvovirus

+; Positive, −; Negative-

**Supplementary Table S4.**

Health records of cats from epidemiological studies conducted in western Japan and detection of viruses in feline pharyngeal swab fluid samples.

| **Cat ID** | **Date of examination** | **Sample collection location** | **Adult / Juvenile** | **Sex** | **Symptom/history of a disease** | **CPE** | **RT-PCR FCV** |
| --- | --- | --- | --- | --- | --- | --- | --- |
| EO-1 | 2022.8.25 | Ozu, Ehime | Juvenile | F | － | − | − |
| EO-2 | 2022.8.25 | Ozu, Ehime | Juvenile | Unknown | Ocular discharge | + | + |
| EO-3 | 2022.8.25 | Ozu, Ehime | Juvenile | M | Ocular discharge | − | − |
| EO-4 | 2022.8.25 | Ozu, Ehime | Juvenile | M | Ocular discharge, diarrhea | − | − |
| EO-5 | 2022.8.25 | Ozu, Ehime | Adult | F | － | + | + |
| EO-6 | 2022.8.25 | Ozu, Ehime | Adult | F | － | − | − |
| EO-7 | 2022.8.25 | Ozu, Ehime | Adult | M | － | − | − |
| EO-8 | 2022.8.25 | Ozu, Ehime | Adult | M | － | + | + |
| EO-9 | 2022.8.25 | Ozu, Ehime | Adult | M | － | + | + |
| EO-10 | 2022.8.25 | Ozu, Ehime | Adult | F | － | + | + |
| O-1 | 2022.11.20 | Osaka | Adult | F | During treatment for hyperthyroidism | − | − |
| O-2 | 2022.11.21 | Osaka | Adult | M | Cystitis, during treatment for bladder stones | − | − |
| O-3 | 2022.11.21 | Osaka | Adult | M | During treatment for renal insufficiency | − | − |
| O-4 | 2022.11.22 | Osaka | Adult | F | － | − | − |
| O-5 | 2022.11.22 | Osaka | Adult | M | Nasal discharge | − | − |
| O-6 | 2022.11.22 | Osaka | Adult | M | Gingivitis, drooling | + | + |
| O-7 | 2022.11.22 | Osaka | Adult | F | Gingivitis, tartar | − | − |
| O-8 | 2022.11.28 | Osaka | Adult | M | Gingivitis, tartar | − | − |
| O-9 | 2022.12.5 | Osaka | Adult | F | Nasal discharge | + | + |
| O-10 | 2022.12.5 | Osaka | Adult | F | Ocular discharge, drooling | − | − |
| O-11 | 2022.12.12 | Osaka | Adult | M | Nasal discharge | − | − |
| O-12 | 2022.12.21 | Osaka | Adult | F | － | − | − |
| O-13 | 2022.12.21 | Osaka | Adult | F | Stomatitis | + | + |
| O-14 | 2022.12.21 | Osaka | Adult | M | Stomatitis | − | − |
| K-1 | 2022.11.14 | Kumamoto | Adult | M | － | + | + |
| K-2 | 2022.11.14 | Kumamoto | Adult | M | － | + | + |
| K-3 | 2022.11.14 | Kumamoto | Adult | F | － | − | − |
| K-4 | 2022.11.14 | Kumamoto | Adult | M | － | − | − |
| K-5 | 2022.11.14 | Kumamoto | Adult | M | － | − | − |
| K-6 | 2022.11.14 | Kumamoto | Adult | M | － | − | − |
| K-7 | 2022.11.14 | Kumamoto | Adult | F | Nasal discharge | − | − |
| K-8 | 2022.11.14 | Kumamoto | Adult | F | － | − | − |
| K-9 | 2022.11.14 | Kumamoto | Adult | M | － | − | − |
| K-10 | 2022.11.14 | Kumamoto | Adult | M | － | − | − |
| K-11 | 2022.11.14 | Kumamoto | Adult | F | － | − | − |
| K-12 | 2022.11.14 | Kumamoto | Adult | M | － | − | − |
| K-13 | 2022.11.14 | Kumamoto | Adult | M | Nasal discharge | − | − |
| K-14 | 2022.11.14 | Kumamoto | Adult | M | Nasal discharge, ocular discharge | − | − |
| K-15 | 2022.11.14 | Kumamoto | Adult | F | － | − | − |
| K-16 | 2022.11.14 | Kumamoto | Adult | F | － | − | − |
| K-17 | 2022.11.14 | Kumamoto | Adult | F | Nasal discharge | − | − |
| K-18 | 2022.11.14 | Kumamoto | Adult | M | Ocular discharge | − | − |
| K-19 | 2022.11.14 | Kumamoto | Adult | M | － | + | + |
| K-20 | 2022.11.14 | Kumamoto | Adult | M | Ocular discharge | − | − |

CPE, cytopathic effect; FCV, feline calicivirus

Adult cats; 1 year and older

M; Male, F; Female

+; Positive, −; Negative

**Supplementary Table S5.**

Amino acid mutations found by comparing the full-length genomes of FCV/Aoshima/Scarlet/2020 and FCV/Aoshima/Scarlet/2023.

| **ORF** | **Protein** | **Mutation** |
| --- | --- | --- |
| ORF1 | NS1 | H20R |
|  | NS2 | N18T, D33E, N78S, A111T, A112T, M165A, V253I |
|  | NS6/7 | V191I, E210D, V251I, S276C, S405C, S630N, S665C |
| ORF2 | LC | I21V, E80D |
|  | VP1 | A8T, L14M, S23N, V116I, N177S, G236D, R274K, D275E, E304K, I306T, A308V, V317N, N318Q, S319N, T322V, S323T, I331T, K334I, E336N, R340K, Q369S, D370G, K372D, S382T, K393G, H394D, E395Q, T396P, G427S, V428I, L472F, H511S, N514K, K530R, S539G, S540T, F541L |
| ORF3 | VP2 | I106N |

**Supplementary Table S6.**

Presence of VS-FCV type amino acid mutations proposed by Brunet et al in FCV isolate (Brunet et al., 2019).

| **Virus strain** | **Symptom** | **438 (Not Polar or Aliphatic)** | **440 (Not Small)** | **448 (Polar or Charged or Not Small or Positive** | **452 (Not Small)** | **455 (Not Charged or Not Negative)** | **465 (Not Hydrophobic or Polar)** | **492 (Small)** | **Total Point** |
| --- | --- | --- | --- | --- | --- | --- | --- | --- | --- |
| UTCVM-NH9 | Upper respiratory tract disease | 1 | 0 | 0 | 0 | 0 | 0 | 1 | 2 |
| ACT7 | Upper respiratory tract disease | 1 | 0 | 1 | 1 | 0 | 1 | 1 | 5 |
| FCV-255 | Pneumonia | 1 | 0 | 0 | 0 | 1 | 0 | 0 | 2 |
| F9 | − (vaccine) | 1 | 0 | 0 | 0 | 0 | 0 | 0 | 1 |
| UTCVM-H1 | Virulent systemic disease | 1 | 1 | 1 | 1 | 1 | 1 | 1 | 7 |
| E1/V2/NSW5 | Virulent systemic disease | 1 | 1 | 1 | 1 | 1 | 1 | 1 | 7 |
| VS-FCV-Ari | Virulent systemic disease | 1 | 0 | 1 | 1 | 0 | 0 | 1 | 4 |
| FCV/Aoshima/Char/2020 | Severe flu-like disease | 1 | 0 | 0 | 0 | 1 | 1 | 0 | 3 |
| FCV/Aoshima/Kotori/2020 | Severe flu-like disease | 1 | 1 | 0 | 0 | 1 | 1 | 1 | 5 |
| FCV/Aoshima/Shiro/2020 | Severe flu-like disease | 1 | 1 | 0 | 0 | 1 | 1 | 0 | 4 |
| FCV/Aoshima/Scarlet/2020 | Severe flu-like disease | 1 | 1 | 0 | 0 | 1 | 1 | 1 | 5 |
| FCV/Aoshima/Hoho/2020 | Severe flu-like disease | 1 | 0 | 0 | 0 | 1 | 1 | 1 | 4 |
| FCV/Aoshima/John/2020 | Severe flu-like disease | 1 | 0 | 1 | 0 | 1 | 1 | 1 | 5 |
| FCV/Ozu/EO-2/2022 | Upper respiratory tract disease | 1 | 1 | 1 | 0 | 1 | 1 | 0 | 5 |
| FCV/Kumamoto/K-1/2022 | Upper respiratory tract disease | 1 | 1 | 0 | 0 | 0 | 0 | 1 | 3 |
| FCV/Osaka/O-6/2022 | Upper respiratory tract disease | 1 | 1 | 1 | 1 | 1 | 1 | 0 | 6 |

For each of the seven amino acid sites proposed by Brunet et al., which are important for distinguishing VS-FCV strains from classical strains, a score of 1 was assigned to strains with VS-FCV-type amino acid characteristics and 0 to those without. The amino acid sites in VP1 as defined by Brunet et al. and the amino acid properties that VS-FCV is expected to have are as follows: 438: Not Polar or Aliphatic; 440: Not Small; 448: Polar or Charged, Not Small, or Positive; 452: Not Small; 455: Not Charged or Not Negative; 465: Not Hydrophobic or Polar; 492: Small.

FCV, feline calicivirus; VS-FCV, virulent systemic feline calicivirus
